# Research on the Mechanisms of Plant Enrichment and Detoxification of Cadmium

**DOI:** 10.3390/biology10060544

**Published:** 2021-06-17

**Authors:** Gui-Li Yang, Meng-Meng Zheng, Ai-Juan Tan, Yu-Ting Liu, Dan Feng, Shi-Ming Lv

**Affiliations:** 1 College of Life Sciences, Guizhou University, Guiyang 550025, China; glyang3@gzu.edu.cn (G.-L.Y.); zmm18073198235@163.com (M.-M.Z.); ajtan@gzu.edu.cn (A.-J.T.); liuyut90@163.com (Y.-T.L.); fengdan1901@163.com (D.F.); 2 State Key Laboratory of Environmental Geochemistry, Institute of Geochemistry, Chinese Academy of Sciences, Guiyang 550081, China; 3 College of Animal Science, Guizhou University, Guiyang 550025, China

**Keywords:** heavy metals, cadmium pollution, phytoremediation, enrichment mechanisms

## Abstract

**Simple Summary:**

Generally, plants undergo a series of oxidative damage and even die after being stressed by cadmium (Cd). Recently, numerous studies about hyperaccumulators that can tolerate and enrich cadmium in the environment have been reported, revealing its potential in restoration of heavy metal pollution. However, there is a lack of systemic understanding of the mechanisms of Cd accumulation and detoxification by hyperaccumulators. Therefore, the purpose of this review is to investigate how these plants absorb, transport, and distribute Cd. The role of plant roots, compartmentalization, chelation, antioxidants, stress, and osmotic adjustment in bioaccumulation of Cd are comprehensively discussed. This review contributes to further understanding the mechanisms of plant enrichment and detoxification of heavy metals.

**Abstract:**

The heavy metal cadmium (Cd), as one of the major environmentally toxic pollutants, has serious impacts on the growth, development, and physiological functions of plants and animals, leading to deterioration of environmental quality and threats to human health. Research on how plants absorb and transport Cd, as well as its enrichment and detoxification mechanisms, is of great significance to the development of phytoremediation technologies for ecological and environmental management. This article summarises the research progress on the enrichment of heavy metal cadmium in plants in recent years, including the uptake, transport, and accumulation of Cd in plants. The role of plant roots, compartmentalisation, chelation, antioxidation, stress, and osmotic adjustment in the process of plant Cd enrichment are discussed. Finally, problems are proposed to provide a more comprehensive theoretical basis for the further application of phytoremediation technology in the field of heavy metal pollution.

## 1. Introduction

With the acceleration of modern industrialisation, the problem of heavy metal pollution has become increasingly prominent [1,2,3]. At present, nearly 20 million hectares of farmland in China are polluted by heavy metals such as mercury, cadmium (Cd), and lead [4]. In 2015, a soil research survey showed that 16.1% of China’s soil and 19.4% of agricultural soil were polluted by heavy metals, of which cadmium pollution (7.0%) was the most serious. The Ministry of Ecology and Environment of China announced in 2019 that cadmium was still the main heavy metal pollutant in the soil [5,6,7].

Cd is a toxic heavy metal that can exist in soil, water, and the atmosphere in various forms [8,9]. Cd^0^ in the atmosphere can become immobilised by combining with iron (Fe) and manganese (Mn) oxides and can also be atmospherically deposited on rain, dust, and snow [10]. Cd in soil and water is usually in an exchangeable state (CdCl_2_ and other water-soluble forms), carbonate-bound state (such as CdHCO_3_^−^), organic-bound state (combined with organic matter in the environment), or residual state (such as H_2_SiO_3_) [8]. Cd in plants is mainly in an inorganic form (such as chloride), Cd-phosphate complex (such as CdHPO_4_), and in the form of binding with pectin and protein. These associations have been confirmed by studies of *Echinodorus osiris Rataj* [11], *Myriophyllum aquaticum* [12], and *Raphanus sativus* L. [13].

Cd can impair animal and plant growth and human health [14,15]. The growth of radish was significantly inhibited when grown in soil containing with 5.0 mg kg^−1^ (dry weight, DW) Cd [13]. The biomass, root length, plant height, and the chlorophyll of *Ceratopteris pteridoides* were reduced when exposed to water containing 20 μM Cd [16]. The length and dry weight of marigold were reduced when grown in soil containing 50 mg kg^−1^ (DW) Cd [17]. Cd from the soil is taken up by crop plants through a migration process and enters the food chain [18,19]. Cd is ingested into the human body through diet and deposited in tissues. Cd-related liver and brain toxicity has been described in humans ingesting a diet containing 1 mg kg^−1^ (body weight)/day or 70 mg/day Cd, respectively [14]. Cadmium can also cause damage to the liver and brain, leading to high blood pressure and even cancer [14,20,21,22,23].

As an abiotic stress factor, Cd can affect the growth and development of plants to varying degrees [14]. When Cd in the environment exceeds a certain concentration, it will stimulate oxidative stress in plants, induce lipid peroxidation, and increase the accumulation of reactive oxygen species (ROS), leading to oxidative damage [20,24,25,26]. In addition, Cd can also cause slower plant growth, decreased chlorophyll content, yellow leaves, and slower photosynthetic rate. Therefore, the growth, development, and physiological and biochemical effects of plants are affected, and high Cd concentration can even cause plant death [16,27,28].

Different plants have different tolerances to Cd. Radish biomass was reduced in the presence of 5.0 mg kg^−1^ (DW) Cd treatment [13]. However, no change in the biomass of *Lantana camara* L. (a hyperaccumulator) has been observed when the Cd lower than 100 mg kg^−1^ (DW), and chlorophyll does not decrease even exposure with 200 mg kg^−1^ (DW) Cd [29]. Among them, hyperaccumulators can over-absorb heavy metals and transport and retain them in the shoots [30,31,32]. Plant species that contain more than 1000 μg of heavy metal per gram (DW) are called hyperaccumulators [26]. Hyperaccumulators have been reported include *Echinodorus Osiris Rataj* [11], *Youngia japonica (L.) DC* [33], *Ceratopteris pteridoides* [16], *Lantana camara* L. [29], *Pterocypsela laciniata* [34], *Sedum alfredii* [35], *Microsorum pteropus* [36], *Taraxacum ohwianum Kitam.* [37], *Siegesbeckia orientalis* L. [38], *Thlaspi caerulescens* [39], and Arabidopsis [40]. Compared with normal plants, hyperaccumulators can not only maintain normal physiological function in a high-concentration heavy metal environment but can also absorb heavy metals to enrich them [12,38]. Compared with physical and chemical methods to control Cd pollution, the bioremediation method using hyperaccumulators has the advantages of low cost and no secondary pollution [31,41,42]. The roots of plants absorb Cd from the environment and then transport it to other tissues through transporters and enrich it inside the plant, thereby limiting the toxicity of Cd to plants and reducing the pollution of Cd in the environment. Therefore, hyperaccumulators show great potential in repairing Cd pollution and have become a research hotspot in the field of heavy metal pollution.

Several published studies focused on the absorption and transport of Cd and the detoxification mechanisms in plants. Shahid et al. [8] reviewed the bioavailability, absorption, transport, and detoxification of Cd in plants in the soil. Ismael et al. [43] summarized the toxicity of Cd to plants and discussed how selenium (Se) reduces the toxic effects of Cd on plants. Riaz et al. [44] further investigated the role of Se and silicon (Si) in reducing the toxicity of Cd in plants and clarified the potential detoxification mechanism of Se and Si to plants. Li et al. [45] summarized the hyperaccumulators reported in China and their tolerance and the effect of Cd on various aspects. However, no study has comprehensively reviewed the absorption, transport, and distribution of Cd in plants or the mechanisms of enrichment and detoxification of Cd by plants.

This article reviews the studies on the uptake, transport, distribution, and enrichment mechanisms of heavy metal Cd in plants and further elucidates the mechanisms of Cd enrichment in plants to promote the advantages of hyperaccumulators in phytoremediation technology for Cd pollution.

## 2. Uptake, Transport, and Distribution of Cd in Plants

### 2.1. Pathways of Cd Uptake by Plants

**Figure 1:**
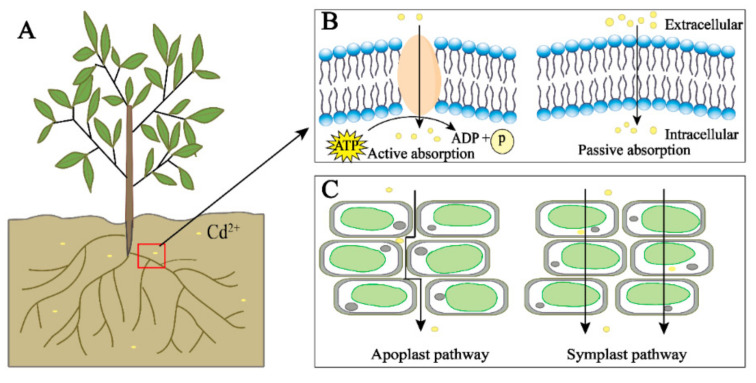
The root uptake of cadmium in plants. Cd (yellow) in the environment can enter plants through plant roots (**A**); when the concentration of Cd is low, ATP is consumed for active transport, and when the concentration is high, it directly enters cells for passive absorption (**B**); Cd is transported between cells by the apoplast and symplast pathways (**C**).

The main way for Cd to enter plants is to be taken up from the soil or water through the roots of the plant, and it can also be taken up from the atmosphere through the leaves of the plant [46,47]. The uptake of Cd by the roots is mainly divided into two steps. First, Cd interacts electrostatically with plant root secretions and negatively charged carboxyl groups on the root cell wall, thereby adsorbing onto plant roots [48,49]. This step is spontaneous and rapid. Then, the Cd adsorbed by the roots passes through the plant cortex by plasma membrane transporters such as natural resistance-associated macrophage protein and is finally absorbed by the plant root cells. This step is achieved by the high mobility and water solubility of Cd. Studies have shown that, as the Cd concentration in the environment increases, the Cd content in plants also increases, but the rate of Cd transport from plant roots to shoots decreases [50]. Cd^2+^ in the environment is absorbed into plants through competition with metal cations such as Cu^2+^, Fe^2+^, Mn^2+^_,_ and Zn^2+^. Therefore, the higher the Cd concentration in the environment, the greater its competitive advantage [51,52,53]. Cd enters the cell through active transport at low concentrations and passive transport at high Cd concentrations (Figure 1). In addition, the content of heavy metal particles in the air in polluted areas is significantly related to the content of heavy metals in plant leaves, which is speculated to be related to atmospheric deposition [54,55]. Heavy metal particles in the air can be deposited on the surface of plants by rain and dust and are directly absorbed into the plant body through the stomata of the leaf epidermis or interact with the cuticle of the epidermis to be absorbed by the plant [56,57]. Although there are two uptake pathways, root absorption is the main pathway of uptake.

### 2.2. Plant Transport of Cd and Involved Transporters

There are two main transport pathways of Cd in plants: the symplast and apoplast pathways. The apoplast pathway is where Cd is transported through the gap between plant cell walls and the gap between the cell wall and the plasma membrane; the symplast pathway is where Cd is transported into the cell through the transporters and then transported between the cells through the plasmodesmata (Figure 1C) [56]. Cd in the environment is actively or passively absorbed by plants, first loaded in the root xylem, and then further transported through the xylem to the plant shoots [58,59]. In plants, Cd can also be efficiently transported from senescent tissues to young tissues through the phloem to facilitate the transport and redistribution of Cd in plants [30].

Plants require the participation of transporters in the process of uptake and transport of Cd, which mainly include yellow-stripe 1-like transporter (YSL) [60,61], zinc-regulated and iron-regulated transporter-like protein (Zrt, Irt-like, ZIP) [62,63], heavy metal ATPase (HMA) [64,65], natural resistance-associated macrophage protein (NRAMP) [66,67], cadmium accumulation in leaf 1 (CAL1) [68,69], and ATP binding cassette transporters (ABC) [70]. Among them, the YSL protein is considered to play an important role in the process of plant transportation of Cd^2+^, Fe^2+^, Fe^3+^, Ni^2+^, Zn^2+^, and other metal chelates, such as the chelate formed by Fe^3+^ and nicotianamine, and the chelate formed by Fe^3+^ and phytosiderophores. The YSL protein is an oligopeptide transporter involved in the transport of metal–nicotianamine complexes [60,61]. The YSL protein (YS1) of corn’s main function is to transport the Fe–phytosiderophore complex needed for corn growth into corn root epidermal cells [71,72]. If the *YSL* gene of maize is mutated, it will lead to the loss of part of the transport function of maize, which is manifested as leaf interveinal chlorosis, so it is named the “yellow stripe” transporter [73]. Das et al. [74] cloned the *BjYSL* gene from the Cd hyperaccumulator, *Brassica juncea*, and *BjYSL6.1*, which was specifically upregulated in shoots after Cd treatment, indicating that the YSL protein is involved in the transport of Cd from roots to shoots. Subsequently, Wang et al. [75] overexpressed the *BjYSL7* gene in tobacco and found that the Cd content in the *BjYSL7* overexpression plants was significantly higher than that of the wild type, the roots were longer, and the root hair development was better, further proving that the BjYSL protein participates in the transport of Cd to shoots. *SnYSL3* was also cloned in another Cd hyperaccumulator, *Solanum nigrum*, and overexpressed in the model plant *Arabidopsis thaliana*. The study found that *SnYSL3* was expressed in the vascular tissue and epidermal cells of *Arabidopsis* roots and stems. *SnYSL3* expression was significantly upregulated after treatment with 100 µM CdCl _2_ for 24 h, and the Cd transport rate of overexpressed *SnYSL3* plants was significantly higher than that of the wild type, which proved that the YSL protein is involved in the uptake and transport of Cd in plants [76]. Although many studies have shown that the YSL protein is involved in the transport of Cd, there are few studies on YSL protein as a whole. In particular, the structure of the YSL transporter is still unclear.

The ZIP family is responsible for the uptake and transport of essential and nonessential metal ions in plants and participates in the uptake and transport of a variety of divalent cations, including Cd^2+^ [62,77]. Most ZIP proteins have eight transmembrane domains, the amino and carboxyl ends of which are located outside the cell [62,78]. The area between the third and fourth domains is called the “variable domain”. In the variable domain, there is a histidine-rich domain that is considered to be part of the cytoplasm and is a metal ion binding site [79]. The mutants lacking this domain have Cd transport ability, and the mutants lacking part of the domain have enhanced transport activity, indicating that the histidine-rich domain is related to Cd transport activity [79]. Grotz et al. [63] cloned *ZIP1*, *ZIP2*, and *ZIP3* from *Arabidopsis thaliana* and expressed the *ZIP* genes in *zrt1* and *zrt2* mutants (*zrt1* and *zrt2* mutants were inhibited in zinc uptake due to lack of high-and low-affinity zinc uptake systems), and the uptake of zinc by yeast was increased after the expression of *ZIP1* and *ZIP3* genes. In addition, Cd can inhibit zinc uptake mediated by *ZIP1, ZIP2*, and *ZIP3*, indicating that Cd can also be taken up from the soil by ZIP transporters and be transported to plants as a substrate of ZIP transporters. Similarly, *VsRIT1* is a *ZIP* gene cloned from *Vicia sativa*. After *VsRIT1* is expressed in *Arabidopsis* and yeast, the transport and accumulation of Cd is significantly increased, while the tolerance of Cd in yeast is reduced [80]. This is consistent with the increase in Cd accumulation after *BcZIP2* expression in *Brassica campestris* L. ssp. *Chinensis* [81], which fully proves that ZIP protein is involved in the process of Cd uptake and transport.

The HMA protein, also called P_1B_–ATPase, belongs to the P-type ATPase gene family. The HMA transporter has eight transmembrane helices. There is a CPX/SPC motif on the sixth, larger transmembrane helix, and it is speculated that there are metal binding domains at the N-terminus and/or C-terminus [65,82,83]. According to the difference in binding specificity with the substrate, the HMA transporter can be divided into two subgroups: the Cu/Ag subgroup, and Zn/Co/Cd/Pb subgroup [50]. Rice [64], *Populus tomentosa* Carr. [84], *Brassica juncea* [85] and *Arabidopsis thaliana* [86], and many other hyperaccumulators contain the HMA Zn/Co/Cd/Pb subgroup. A repeating region near the C-terminus in *Brassica juncea* is named *BjHMA4R* [85]. Cloning and expressing *BjHMA4R* in yeast is beneficial in improving the tolerance to Cd and the accumulation of Cd, which was confirmed by the analysis of the C-terminal region of OsHMA3 transgenic *Arabidopsis thaliana* [86]. Arabidopsis *hma4* mutants and *hma2hma4* mutants were more sensitive to Cd and had significantly inhibited root growth. The *hma2hma4* mutant increased Cd accumulation in the roots, and the transport of Cd from roots to shoots was 2–3% of the wild type [87], which is consistent with the result that the *Athma4* mutant transport rate decreased by at least 50% [88]. In the two overexpressed *HMA Arabidopsis* lines modified by the 35S strong promoter, Cd accumulation in rosette leaves was three times that of the wild type. The Cd concentration in the roots and shoots of the overexpressed rice *OsHMA3* strain was higher than that of the control group, and the roots showed higher Cd content at low and high Cd culture concentrations. This is consistent with the conclusion that Cd accumulation in *PtoHMA5* transgenic tobacco leaves increased by 25.04% and the transport rate increased by 16.7–43.25% [84]. In addition, immunostaining studies have shown that cucumber *CsHMA3* and *CsHMA4* were expressed in the tonoplast and plasma membrane of cucumber root cells, respectively [89], and cucumber *CsHMA3* and *CsHMA4* were expressed in the vacuole membrane and plasma membrane of cucumber root cells, respectively, indicating that HMA protein exists in the root cell membrane and tonoplast membrane and plays an important role in the uptake of Cd from roots and the transport of Cd to shoots.

The NRAMP protein is a family of membrane-integral transport proteins. NRAMP1 is the first protein discovered in the NRAMP family and participates in the process of macrophage resistance to bacterial infection by transporting Fe^2+^ [90]. *Sedum alfredii SaNRAMP6* [91] is expressed on the plasma membrane of epidermal cell protoplasts. After heterologous expression in yeast, the Cd content increased, and the same was true after transfer to *Arabidopsis*. This is consistent with the increase in Cd concentration in roots, stems, leaves, and whole plants after *TtNRAMP6* overexpression in *Arabidopsis thaliana* [92], and the sensitivity to Cd and the increase in Cd content after the heterologous expression of *TcNRAMP3* in *Thlaspi caerulescens* [93]. In addition, Wu et al. [94] found that *HvNRAMP5* was mainly expressed in root epidermal cells, and the expression of the root tip was higher than that of the root base. Further research by Tiwari et al. [95] showed that the root endothelial layer and pericyll cells were the main sites of rice *OsNRAMP1* expression in *Arabidopsis*, and *NcNRAMP1* has been shown to participate in the process of Cd passing through the endothelial plasma membrane [66]. The presence of NRAMP protein is beneficial to the xylem parenchyma of plant roots to load Cd and transport it to young plant parts in the phloem [30].

In the process of heavy metal uptake and transport, the participation of transporters and their importance (Table 1) has received extensive attention from scholars. Many transporters, such as YSL, ZIP, HMA, and NRAMP, that participate in the uptake and transport of Cd have been studied. However, the molecular mechanism of the specific binding of the abovementioned transporter to metal ions and the relationship between the structure and function of the transporter are still unclear. These are essential in elucidating the mechanisms of Cd uptake and transport in plants.

### 2.3. Distribution and Subcellular Distribution of Cd in Plants

Heavy metals such as Cd are absorbed from the environment by these transporters into plant roots and then transported to shoots, where they are distributed to various tissues of the plant. The distribution of Cd in plants varies according to plant species and varieties (Table 2). In different types of daily vegetables, the order of Cd accumulation capacity (which is termed the bioconcentration factor, corresponding to the ratio of the Cd content in vegetables and soil) is leafy vegetables (18.9%) > solanum fruits (4.9%) > legumes (2.1%) > melons (1.6%) > tubers (1.4%) [110]. The distribution of Cd in different plant tissues (Table 2) usually shows that the accumulation of Cd in the roots of plants is the largest, and the accumulation of Cd in shoots is less than that in roots, which is related to the retention of Cd by plant roots [88]. In Monashree’s research on *Ceratopteris pteridoides* [16], the accumulation of Cd is as follows: root (1316.34 mg kg^−1^, DW) > leaf (191.38 mg kg^−1^, DW) > stem (186.19 mg kg^−1^, DW). The results are similar to those for the Cd hyperaccumulator, *Microsorum pteropus* [111]. In another *Camellia sinensis* study, the accumulation of Cd was shown as stem (0.52 mg kg^−1^, DW) > leaves (0.12 mg kg^−1^, DW) > new shoots (0.06 mg kg^−1^, DW) [112]. Similar results appeared in soybeans [113]. In a study on tobacco [114] and *Solanum nigrum* [115], the content of Cd in leaves was significantly higher than in roots and stems. It is speculated that the Cd in these plants is mainly concentrated in the roots at low Cd concentrations. When the environmental Cd content increases, the uptake and distribution mode of the plant is adjusted, the Cd uptake of the whole plant increases, and Cd is enriched in the leaves. The Cd concentration in the shoots and roots of *Youngia japonica *(L.) DC under the treatments of 10 mg kg^−1^ Cd (DW) and 30 mg kg^−1^ (DW) Cd in soil was similar. Under 120 mg kg^−1^ (DW) Cd treatment in soil, the Cd concentration of the shoots reached 314.29 mg kg^−1^ (DW), which was significantly higher than that of roots (252.51 mg kg^−1^, DW) [33], which also supports this hypothesis.

Moreover, the distribution of Cd at the plant subcellular level is uneven (Table 2). In Monashree’s research, the overall Cd accumulation was as follows: cell wall (28–69%) > organelles (14–44%) > soluble fractions (6–46%). However, Cd distribution in the same subcellular tissue of different plant differs. The difference is that, under the treatments of 10 μM, 20 μM, and 40 μM Cd (hydroponics), the content of Cd in the soluble fractions of the leaves is higher than that in the organelles [16]. In Wang’s study [9], the subcellular distribution (the ratio of total Cd in different subcellular structure to the total Cd in the whole cell) of Cd in soybean roots at 23 μM and 45 μM levels was as follows: cell wall (53.4–75.5%) > soluble fraction (15.8–40.4%) > organelles (2.0–14.7%), while the shoot soluble fraction (39.3–74.8%) > cell wall (16.0–52.0%). Similar to the subcellular distribution in soybean roots, the subcellular distribution of Cd in the Cd hyperaccumulator, *Solanum nigrum,* was cell wall (62–66%) > soluble fraction (26–32%) > organelle (5–7%) [115]. This indicates that the root cell wall is the main subcellular location for plants to store Cd, which is achieved by the retention effect of roots, followed by Cd transported to shoots and stored in the vacuole in the form of soluble fractions. *Thlaspi caerulescens* [39], a Cd hyperaccumulator, can accumulate high concentrations of Cd and Zn in the leaves. Further research found that 91% of the Cd in leaves was stored in protoplasts and located in vacuoles. This may be related to the fact that Cd is stored in vacuoles of plant cells through chelation and compartmentalisation, including heavy metal ions interacting with ligands.

The distribution and subcellular distribution of Cd in plants are uneven due to differences in plant species and varieties. At present, the distribution and subcellular distribution of Cd in plants as physiological and biochemical characteristics have been widely studied by scholars, but there is no unified understanding of whether the difference in its distribution is related to the significance of enrichment and tolerance.

## 3. The Mechanisms of Plant Enrichment of Cd

**Figure 2:**
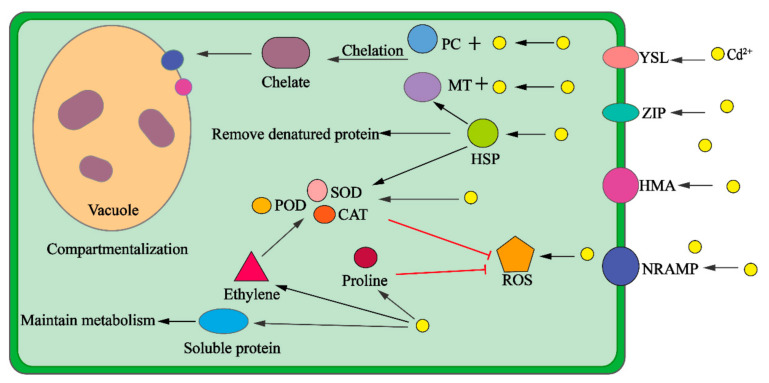
The mechanisms of compartmentalization, chelation, antioxidation, stress, and osmotic adjustment in plants. PC: phytochelatin; MT: metallothionein; HSP: heat shock protein; ROS: reactive oxygen species; SOD: superoxide dismutase; POD: peroxidase; CAT: catalase; YSL: yellow-stripe 1-like transporter; ZIP: zinc-regulated and iron-regulated transporter-like protein; HMA: heavy metal ATPase; NRAMP: natural resistance-associated macrophage protein.

Under Cd stress, plants show a series of physiological responses to achieve detoxification and enrichment of heavy metals (Figure 2). Current studies have shown that the main response mechanisms of plants under Cd stress are the retention effect of roots, compartmentalisation, chelation, antioxidation, stress, and osmotic adjustment [43,118]. Among them, root function, compartmentalisation, and chelation in plants are the dominant factors in the process of Cd enrichment. Other plant response mechanisms also play an important role in achieving Cd enrichment in plants, regulating physiology, and maintaining life activities.

Cd^2+^ enters the cell by combining with transporters such as YSL, ZIP, HMA, and NRAMP and with small molecules such as phytochelatin (PC) to form chelates, which are transported to the vacuole to achieve compartmentalisation. Cd^2+^ can stimulate the production of ROS, cause oxidative stress, and stimulate the plant’s antioxidant defence system. Antioxidant enzymes, such as superoxide dismutase (SOD), peroxidase (POD), and catalase (CAT), eliminate reactive oxygen species (ROS) through increased activity. Heat shock protein (HSP) induced by stress can upregulate antioxidant enzyme activity, upregulate MT expression, and remove denatured proteins. Appropriate ethylene synthesis can also regulate antioxidant activity. The increase in proline content is conducive to the elimination of ROS, and an increase in soluble protein content is conducive to maintaining cell stability and cell metabolism.

### 3.1. The Role of Plant Roots

Plant roots serve as the first barrier for Cd entering plants. The root secretions and the cell wall of root epidermal cells play an important role in limiting Cd uptake and Cd accumulation in the roots [58]. Root exudates can form complexes or precipitates with external Cd, thereby retaining Cd on the root epidermis [119]; organic acids in different types of root exudates also have different adsorption effects on Cd in the soil, so that Cd in the soil enters the plant roots in different chemical forms. Therefore, the bioavailability of Cd in the soil environment can be conversed by changing the root exudates [119]. Lowering the pH increases the solubility of Cd compounds in the soil and, hence, the bioavailability of Cd^2+^. An appropriate concentration of root exudates can promote the transfer of Cd from roots to leaves for accumulation in the leaves [119]. The root cell wall is mainly composed of cellulose, hemicellulose, and pectin, which are rich in hydroxyl, thiol, and carboxyl functional groups. These functional groups can bind to Cd^2+^ so that Cd can be retained in the root cell wall [117,120]. Cd in roots mainly accumulates in the cortical tissues of root tips and root hairs. Due to the existence of the Casparian strip in the endothelium, the transport of Cd to the central pillar is further prevented [121]. Wang et al. showed that Cd had the highest content in root cell walls, and the retention effect of the cell wall on Cd greatly reduced the damage of Cd to plant cells [9]. Cheng et al. [37] found that a large amount of Cd accumulated in the xylem vessel and xylem cell wall of the Northeast Dandelion root system, indicating that one of the important reasons for *Taraxacum ohwianum* Kitam. to tolerate Cd stress was the retention of the root cell wall to Cd. However, the abovementioned studies have not conducted in-depth studies on how plants regulate the retention of Cd in the roots and the restriction of Cd uptake. It is necessary to reveal the mechanisms by which plant roots absorb Cd and of Cd enrichment.

### 3.2. Compartmentalization and Chelation

After heavy metal ions enter the plant, they can be chelated or even precipitated by metallothioneins (MT), phytochelatins (PC), glutathione, oxalic acid, citric acid, and other small molecules in the plant cytoplasm and vacuoles, thus reducing their toxicity [122,123]. MT is a small protein that is rich in cysteine. It is produced in response to heavy metal ions entering the plant [124,125]. The study found that the Cd tolerance and accumulation ability of yeast enhanced after inserting rice *MT1e* [126], which was consistent with the results of Gu et al. [127], and Chen et al. [128]. The binding of heavy metal ions to the cysteine-rich regions may be one of the mechanisms that plants use for detoxification [129]. PC is a type of sulfhydryl polypeptide containing cysteine, glutamic acid, and glycine synthesised by plants after being stressed by heavy metals [130]. It was first extracted from *Rauvolfia serpentina* and identified by Grill et al. [131]. It was found that it is not the same substance as MT. In the presence of Cd^2+^, PC synthesised combines with Cd^2+^ to form a complex and is then transported by transporters or transported through vesicles to inactive organelles (mainly vacuoles) for compartmentalisation [132,133,134].

Plants transport heavy metals that enter the plant to inactive areas such as cell walls and vacuoles, retain them, and reduce the fluidity of heavy metals by isolating them. This mechanism is called compartmentalisation [40,135]. Vacuoles are the main sites for the storage of heavy metal ions in hyperaccumulators. Vacuole compartmentalisation is one of the mechanisms by which plants enrich and tolerate Cd and is also an important criterion for screening hyperaccumulators [40]. The transport mechanism of vacuole compartmentalisation is divided into two types: transport directly through the transporter on the vacuole, and transport through the vesicle in the cytoplasm and the vacuole membrane [136]. Allan et al. [137] found that Cd easily combines with amino, carboxyl, hydroxyl, and other coordination groups in cell wall proteins and polysaccharides to realise the cell wall compartmentalisation of Cd. When the Cd^2+^ accumulation in the cell wall is higher enough, Cd is then transported to the vacuole. Cd can combine with proteins, organic acids, sugars, and other organic substances in the vacuole to form macromolecular compounds through chelation, thereby reducing the toxicity of Cd [138,139]. Wei et al. [140] found that, after the heavy metals Cd and Cu entered the *Eucalyptus* cells, they were transported to the soluble fractions to reduce damage to the organelles. This proves that the compartmentalisation of soluble fractions is an important mechanism for *Eucalyptus* to accumulate and tolerate Cd [141,142]. Tian et al. showed that the main Cd enrichment sites in *Sedum alfredii* were the cortex and mesophyll tissue, and most of them were located in the vacuole [143], which is consistent with the findings of Huang et al. [117] and Zhang et al. [11].

### 3.3. Antioxidant System

Reactive oxygen species (ROS) play an important role in controlling plant growth, the abiotic stress response, system signal transmission, programmed cell death, and plant development [144]. When the content of ROS in plants increases, the accumulated ROS leads to membrane lipid peroxidation, cell membrane rupture, electrolyte leakage, and DNA loss, which affect the normal physiological and biochemical functions of cells [145]. As ROS levels increase, the antioxidant defence system in plants is activated. As an important part of the antioxidant defence system, antioxidant enzymes in plants can help eliminate ROS and reduce plant damage [26,146]. These enzymes include superoxide dismutase (SOD), catalase (CAT), peroxidase (POD), glutathione peroxidase (GSH-Px), and ascorbate peroxidase (APX). Cd induces ROS production by stimulating NADPH oxidase activity, replacing essential cations at specific binding sites, and inhibiting enzyme activity [147].

Once the plant is stressed by Cd, the plant will adjust the activity of these antioxidant enzymes accordingly, but this change will vary depending on the plant species, the length of exposure to heavy metals, and the growth stage of the plant [148]. Upon exposure to low Cd concentrations in *Ceratopteris pteridoides* [16], the activities of SOD, CAT, and POD are upregulated and increase with the addition of Cd, which is consistent with results for *Vallisneria natans* [27], *Solanum nigrum* L. [115], perennial ryegrass [149], *Lantana camara* L. [29], and *Calendula calypso* [17]; this might be caused by the increase in antioxidant enzyme synthesis under the stimulation of ROS. However, there is a critical value of Cd concentration (70–120 mg kg^−1^, DW), beyond which the antioxidant enzyme activity no longer increases or even decreases, indicating that the ROS content in the plant exceeds the antioxidant enzyme scavenging ability. The rate of antioxidant enzyme synthesis will also be affected and cannot resist oxidative damage under high Cd stress [33,34,116,150]. Lian et al. [151] found that wheat seedlings exposed to Cd increased the activity of CAT and POD but decreased the activity of SOD, which may be caused by the decrease in Fe and Mn plasma concentrations that play a key role in the synthesis of SOD [152]. SOD enzyme activity in *Narcissus tazeta* var. *chinesis* roots showed an initial decline and then an upward trend with increasing Cd concentration. Low concentrations cause oxidative stress and increase with the addition of Cd. High concentrations may lead to the accumulation of ROS, thereby inducing increased SOD production [153]. Yang et al. [154] found that, under a certain concentration of Cd stress, the enzyme activities of CAT, SOD, and POD in tolerant crops, such as winter wheat and other gramineous plants, increased with increasing Cd concentration, while the enzyme activities in plants with poor tolerance, such as cucumbers, decreased. This proves that the increase in antioxidant enzyme activity is directly related to the improvement of plant tolerance. Although antioxidant enzymes such as SOD, CAT, and POD can regulate their activity to eliminate ROS to protect plants within a certain concentration range, the activity of these antioxidant enzymes will still be inhibited at high Cd concentrations. These results have been confirmed in eggplant [115], and *Taraxacum ohwianum* *Kitam.* [37]. Some hyperaccumulators can still maintain high enzyme activity in the presence of high concentrations of Cd (≥100 mg kg^−1^, DW), such as *Lantana camara* L. [29], Solanum *nigrum* L. [115], *Youngia japonica (L.) DC* [33], and *Pterocypsela laciniata* [146]. Therefore, the activity of antioxidant enzymes is considered an important indicator of the ability of plants to tolerate Cd stress.

### 3.4. Stress

When plants are in an environment that subjects them to stresses such as high temperature or the presence of heavy metals, plants begin to synthesise heat shock proteins (HSPs), chitinases, and pathogen-related proteins [155,156]. HSP maintains normal cell metabolism by upregulating the activity of ROS scavenging enzymes (including antioxidant enzymes), reducing ROS accumulation, and removing denatured proteins. It can also bind to cytoplasmic phosphorylated proteins to regulate antioxidant enzyme activity, induce melatonin production, and upregulate MT expression, thereby improving the ability of cells to tolerate Cd [157,158,159], which may be related to the DNA-binding domain in the heat shock transcription factor, HsfA4a [160]. In addition to the synthesis of HSP, plants can also induce ethylene production under Cd stress. Liu et al. [161] and Schellingen et al. [162] both confirmed this, and further found that Cd transported from roots to shoots can increase the ethylene content of shoots. Cd stress can upregulate the expression of 1-aminocyclopropane-1-carboxylic acid (ethylene synthesis precursor) synthase to induce ethylene synthesis, and further regulate the growth inhibition of plant roots by regulating XTH33 and LSU1 mediated by the transcription factor, EIN3 [163,164]. In addition, ethylene can control the SOD content by increasing the activity of SOD isoenzymes, but excessively high ethylene levels inhibit the activities of antioxidant enzymes such as CAT and APX [165,166]. Therefore, it is possible to strengthen the antioxidant defence ability by maintaining an appropriate level of ethylene, thereby increasing the plant’s ability to enrich and tolerate cadmium.

### 3.5. Osmotic Adjustment

After Cd stress, plants can also increase the cell osmotic potential by changing the content of osmotic adjustment substances, including proline, soluble protein, soluble sugar, and small molecular organic acids, thereby eliminating the toxic effect of Cd on plants [167,168]. Proline is an osmotic regulator that exists in a free state, which not only protects the integrity of proteins and prevents enzyme dehydration and inactivation but also scavenges ROS free radicals [169]. Zouari et al. [170] found that the addition of exogenous proline (20 mM) to date palms increased antioxidant enzyme activity and reduced the oxidative damage caused by Cd in plants. The same result was observed in *Olea europaea. L* [171]. *Solanum nigrum* L. [115] and *Parthenium hysterophorus* L. [172] significantly increased the proline content under Cd stress, which proved that proline participates in osmotic regulation under Cd stress. In addition, Zhou, et al. [173] found that the soluble sugar content after Cd treatment was significantly higher than that of the control group, which was consistent with the results of Rady et al. [174]. By increasing the content of soluble protein and soluble sugar in plants, the intracellular osmotic potential can be increased, and the intracellular water content can be maintained, which is helpful in alleviating electrolyte leakage caused by oxidative stress and maintaining the normal physiological function of cells [175,176]. However, the increase in soluble protein content in soybean endosperm is accompanied by a decrease in soluble protein content and soluble sugar content in the radicle [177], which might be related to the inhibitory effect of Cd on the activities of hydrolytic enzymes, including α-amylase, β-amylase, acid phosphatase, and alkaline phosphatase [178]. This suggests that complex osmotic regulatory networks exist in plants that can regulate cell osmotic potential through a series of complex responses to Cd stress to reduce the toxic effects of Cd on plants.

## 4. Conclusions and Outlook

As a widespread pollutant in the environment, Cd not only affects the physiological and biochemical functions of plants but can also be ingested by the human body through the biological chain to have a serious impact on health. Cd in the environment can interact with plant root cell walls or root exudates to be adsorbed on roots, enter plant roots through active or passive absorption, be transported by symplast pathway or apoplast pathway, and transport to shoots through the xylem. Plants use root retention to restrict Cd from being transported to shoots [121]. If Cd^2+^ enters the cell, it combines with small proteins and peptides such as MTs and PCs to attenuate the toxicity of Cd. Cd can be compartmentalised to reduce fluidity and achieve intracellular enrichment. In this process, Cd is combined with YSL, ZIP, HMA, NRAMP, and other transporters and is finally distributed to various tissues of the plant, mainly in the cell wall of plant roots and the vacuole of shoots. As an abiotic stress factor, Cd can stimulate the antioxidant defence system of plants. In response to the oxidative damage caused by Cd, plants reduce ROS by regulating the activity of antioxidant enzymes and synthesising HSP. The synthesis of appropriate levels of ethylene can help improve the antioxidant defence capabilities. In addition, osmotic adjustment substances such as proline, soluble protein, and soluble sugar can also reduce the oxidative damage caused by Cd to plants and exert detoxification effects to further enhance the enrichment of Cd in plants.

The use of hyperaccumulators to reduce Cd pollution is a promising phytoremediation method. Many scholars have obtained important research results on the molecular mechanisms of Cd uptake, distribution, and transport, as well as the molecular mechanism of plant enrichment and tolerance to Cd. However, there are still many problems that have not been resolved, such as the significance of the difference in the distribution of Cd in plants for plants to adapt to the environment and maintain their physiological functions; the chemical, morphological changes of Cd in the process of transport and the molecular mechanism of its binding to transporters; the regulatory mechanism of compartmentalisation and chelation; the regulatory mechanism of antioxidant enzyme activity under Cd stress; and the osmotic regulatory mechanism under Cd stress. In the process of Cd absorption and transport, the structure and morphology of the transporter changes, as do the interactions between different proteins. If the above problems can be solved, research on the mechanism of Cd enrichment in plants will take a big step forward, and phytoremediation technology can be used to treat heavy metal pollution.

## Figures and Tables

**Table 1:** Statistics of transporters in different plants.

Species	Transporter	Metal Ions	Refs
*Brassica juncea*	BjYSLs BjNRAMPs	Cd^2+^	[74]
	BjYSL7	Fe^2+^, Cd^2+^, Ni^2+^	[75]
	BjHMA4	Cd^2+^	[85]
*Arabidopsis*	AtHMA4	Zn^2+^, Cd^2+^	[88]
	HMA2, HMA4	Cd^2+^	[88]
	ZIP1, ZIP2, ZIP3, ZIP4	Zn^2+^	[63]
*Arabidopsis halleri*	AhZIP6	Zn^2+^, Cd^2+^	[96]
*Oryza sativa* L.	OsHMA1, OsHMA 2, OsHMA3	Zn^2+^, Cd^2+^	[64,86,97,98]
	OsNRAMP1, OsNRAMP5	Cd^2+^, As^3+^, Mn^2+^	[63,95,97]
	OsZIP1, OsZIP3	Cu^2+^, Zn^2+^, Cd^2+^	[99,100]
*Thlaspi caerulescens*	TcNRAMP3	Fe3+, Cd^2+^	[93]
	ZNT1, ZNT5 ZIP	Zn^2+^, Cd^2+^	[101]
*Sedum alfredii*	SaNRAMP6	Cd^2+^	[91]
*Sedum plumbizincicola*	SpHMA1, SpHMA3	Cd^2+^	[35,102]
*Brassica campestris* ssp. *chinensis*	HMA2, HMA4, BcGSTU	Cd^2+^	[81,103]
	BcNRAMP5	Cd^2+^	[104]
	BcIRT1, BcZIP2	Cd^2+^, Mn^2+^, Zn^2+^, Fe^2+^	[105]
*Hordeum vulgare*	HvNRAMP5	Mn^2+^, Cd^2+^	[94]
	HvYS1	Fe3+	[106]
*cucumber*	CsHMA3, CsHMA4	Pb^2+^, Zn^2+^, Cd^2+^	[89]
*Solanum nigrum*	SnYSL3	Fe^2+^, Cu^2+^, Zn^2+^, Cd^2+^	[76]
*Populus tomentosa Carr.*	PtoHMA5	Cd^2+^	[84]
*Brassica napus* L.	BnaHMA4c	Cd^2+^	[107]
*Vicia sativa*	VsRIT1	Cd^2+^, Fe(EDTA-Fe), Zn^2+^	[80]
*Triticum turgidum* L. ssp. *turgidum*	TtNRAMP6	Cd^2+^	[92]
*Triticum polonicum* L.	TpNRAMP5	Cd^2+^, Co^2+^, Mn^2+^	[108]
*Nicotiana tabacum*	NtZIP1	Cd^2+^, Zn^2+^	[50]
	NtNRAMP5	Mn^2+^, Cd^2+^	[109]

Refs: references.

**Table 2:** Distribution and subcellular distribution of cadmium in different plants.

Plant	Distribution (mg kg^−1^, DW)				SD (%)			BCF	TF	Refs
	Root	Leaf	Stem	Shoot	CW	OE	SF			
*Ceratopteris pteridoides*	1316.34	191.38	186.19		28–69	14–44	6–46	27.99–570.75	0.10–0.14	[16]
*Lantana camara* L.	293.4	423.06	392.37	301.78				1.32–3.14	1.04–1.41	[29]
*Myriophyllum aquaticum*		122.530	111.828		24.92–38.57	0.97–12.04	57.40–66.25			[12]
*Calendula calypso*	165			78				2.7–4.14	0.46–0.50	[17]
*Koelreuteria paniculata*	8.48	4.04	2.31		45–77	2–11	20–45	0.052–0.318	0.464–0.705	[116]
*Echinodorus Osiris Rataj*	2742.95			502.97	8.44–25.62	−22.07	69.49–88.39	>1	0.18	[11]
*Raphanus sativus* L.	3.52–4.94			4.66–6.86	18–49	15–20	36–51	1.64–2.36	1.32–1.38	[13]
*Morus alba* L.	31.6	8.57	15		31–77	3–13	16–66	0.10–0.35	0.12–0.27	[117]

SD: subcellular distribution; BCF: bioconcentration factor; TF: transport factor; CW: cell wall; OE: organelle; SF: soluble fraction; Refs: references.
